# Regulatory Connections Between the Cyanobacterial Factor PipX and the Ribosome Assembly GTPase EngA

**DOI:** 10.3389/fmicb.2021.781760

**Published:** 2021-12-09

**Authors:** Carmen Jerez, Paloma Salinas, Antonio Llop, Raquel Cantos, Javier Espinosa, Jose I. Labella, Asunción Contreras

**Affiliations:** Departamento de Fisiología, Genética y Microbiología, Facultad de Ciencias, Universidad de Alicante, Alicante, Spain

**Keywords:** protein interaction, PII, *Synechococcus elongatus*, bacterial two hybrid, phenotypic analysis, confocal microscopy, nitrogen regulation network

## Abstract

Cyanobacteria, phototrophic organisms performing oxygenic photosynthesis, must adapt their metabolic processes to important environmental challenges, like those imposed by the succession of days and nights. Not surprisingly, certain regulatory proteins are found exclusively in this phylum. One of these unique proteins, PipX, provides a mechanistic link between signals of carbon/nitrogen and of energy, transduced by the signaling protein PII, and the control of gene expression by the global nitrogen regulator NtcA. PII, required for cell survival unless PipX is inactivated or downregulated, functions by protein–protein interactions with transcriptional regulators, transporters, and enzymes. PipX also functions by protein–protein interactions, and previous studies suggested the existence of additional interacting partners or included it into a relatively robust six-node synteny network with proteins apparently unrelated to the nitrogen regulation system. To investigate additional functions of PipX while providing a proof of concept for the recently developed cyanobacterial linkage network, here we analyzed the physical and regulatory interactions between PipX and an intriguing component of the PipX synteny network, the essential ribosome assembly GTPase EngA. The results provide additional insights into the functions of cyanobacterial EngA and of PipX, showing that PipX interacts with the GD1 domain of EngA in a guanosine diphosphate-dependent manner and interferes with EngA functions in *Synechococcus elongatus* at a low temperature, an environmentally relevant context. Therefore, this work expands the PipX interaction network and establishes a possible connection between nitrogen regulation and the translation machinery. We discuss a regulatory model integrating previous information on PII–PipX with the results presented in this work.

## Introduction

Cyanobacteria, phototrophic organisms that perform oxygenic photosynthesis, constitute an ecologically important phylum that is responsible for the evolution of the oxygenic atmosphere and are the main contributors to marine primary production (Blank and Sánchez-Baracaldo, 2010). Their photosynthetic lifestyle and ease of cultivation make them ideal production systems for several high-value compounds, including biofuels (Khan and Fu, 2020). Cyanobacteria have developed sophisticated systems to maintain the homeostasis of carbon/nitrogen (reviewed by Zhang et al., 2018; Forchhammer and Selim, 2020), the two most abundant elements in all living forms. Therefore, understanding the regulatory mechanisms affecting their metabolic balance is of paramount importance from the biotechnological as well as the environmental point of view.

Cyanobacteria can use different nitrogen sources that are first converted into ammonium and then incorporated *via* the glutamine synthetase–glutamate synthase cycle into carbon skeleton 2-oxoglutarate (2-OG) for the biosynthesis of amino acids and other N-containing compounds. The 2-OG, a universal indicator of the intracellular carbon-to-nitrogen balance (Senior, 1975; Huergo and Dixon, 2015), appears to be particularly suitable for this role in cyanobacteria because the lack of 2-OG dehydrogenase results in the accumulation of 2-OG during nitrogen starvation (Stanier and Bazine, 1977). A role as an antioxidant agent involved in reactive oxygen species (ROS) homeostasis has also been proposed for 2-OG in cyanobacteria (Robles-Rengel et al., 2019).

In bacteria and plants, 2-OG is sensed by the widely distributed and highly conserved signal transduction protein PII, encoded by *glnB*, which forms a highly stable homotrimer with three 2-OG binding sites. PII regulates the activity of proteins involved in nitrogen metabolism by direct protein–protein interactions (Selim et al., 2020). The first two PII receptors identified in cyanobacteria (Burillo et al., 2004; Espinosa et al., 2006) were detected using the yeast two-hybrid system (Fields and Song, 1989) to search for proteins interacting with PII in *Synechococcus elongatus* PCC7942 (hereafter *S. elongatus*). One of the identified proteins was the enzyme N-acetyl-glutamate-kinase, which catalyzes the first key regulatory step of arginine biosynthesis. In cyanobacteria and plant chloroplasts, this enzyme is activated by PII (Burillo et al., 2004; Heinrich et al., 2004; Llacer et al., 2007; Laichoubi et al., 2011; Chellamuthu et al., 2013). The other one was PipX (*P*II *i*nteracting *p*rotein *X*), a small and previously unrecognized protein of 89 amino acids with an N-terminal tudor-like domain (TLD/KOW) and two consecutive C-terminal helixes (Llácer et al., 2010).

PipX was also found as a prey in yeast two-hybrid searches with NtcA, the global transcriptional regulator involved in nitrogen assimilation in cyanobacteria (Herrero et al., 2001; Esteves-Ferreira et al., 2018). A subsequent work confirmed the functional and structural details of the PipX–PII and PipX–NtcA interactions, revealing that three PipX monomers assemble as trimers on the surface of trimeric PII, while two independent PipX monomers bind to dimeric NtcA (reviewed by Forcada-Nadal et al., 2018). PipX stabilizes the conformation of NtcA that is transcriptionally active and probably helps the local recruitment of RNA polymerase. Since TLD/KOW of PipX provides the contacts with both NtcA and PII, PII sequestration of PipX at low 2-OG renders PipX unavailable for NtcA binding and activation, reducing the expression of NtcA-dependent gene targets (Espinosa et al., 2007, 2009, 2010; Llácer et al., 2010; Zhao et al., 2010; Laichoubi et al., 2012). Complex formation with PipX increases the affinity of PII for ADP, and conversely, the interaction between PII and PipX is highly sensitive to fluctuations in the ATP/ADP ratio (Zeth et al., 2014). Thus, PipX swapping between PII and NtcA links PII signaling with NtcA-regulated gene expression.

Additional “guilty by association” approaches have extended the physical and functional networks of PipX. Yeast three-hybrid searches with PipX–PII as bait resulted in the identification of the cyanobacterial transcriptional regulator PlmA as an interacting protein (Labella et al., 2016), while co-expression and synteny approaches functionally connected PipX with PipY, a conserved pyridoxal phosphate-binding protein involved in amino/keto acid and pyridoxal phosphate homeostasis (Labella et al., 2017, 2020a; Tremiño et al., 2017; Cantos et al., 2019). Given that in this phylum most signaling proteins are encoded in monocistronic units, we took the “guilty by association” principle one step further to look for genes that, independently of their operon structures, are closely associated with PipX in cyanobacterial genomes and may thus be functionally connected. For this, we constructed a cyanobacterial linked genome (CLG) (Labella et al., 2020b), a database generated based on the conservation of gene neighborhoods across cyanobacterial species, which can be easily accessed through an interactive platform^1^. The CLG appears to be particularly informative in the context of genes that are specifically related to the photosynthetic lifestyle, such as genes found exclusively in cyanobacteria or cyanobacteria and chloroplasts (discussed in Tiruveedula and Wangikar, 2017).

**FIGURE 1:**
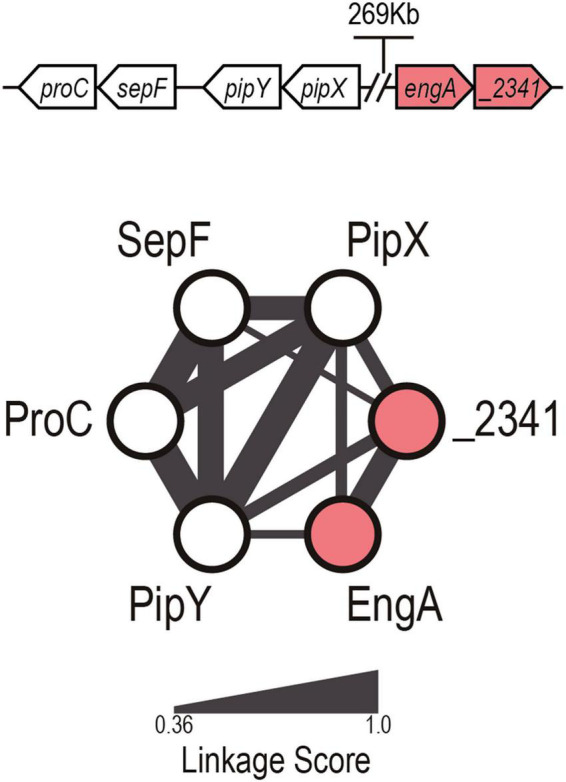
PipX default cyanobacterial linked genome network. Illustration of the dCLG network of PipX, with proteins and predicted relationships represented as nodes and edges, respectively. The edge width is proportional to the linkage score (Labella et al., 2020b). The genomic organization of the genes in *Synechococcus elongatus* is shown at the top (not to scale). Known and previously unnoticed proteins connected to PipX by gene synteny are shown as white and red nodes, respectively.

There is no synteny between the genes encoding PipX and any other of the proteins for which binding has been shown (PII, NtcA, and PlmA). The CLG default outcome (dCLG) for PipX (Figure 1) contains three of its already known (downstream) neighbors in the *S. elongatus* chromosome (*pipY*, *sepF*, and *proC*) and two other previously unconnected genes: *synpcc7942_2340* (*engA*) and *synpcc7942_2341* (a putative T component from an energy-coupling factor transporter). Except for PipX, which is only found in cyanobacteria, all network components have homologs outside this phylum. So far, the only clearly established and reported connection within the cluster concerns the *pipX-pipY* genes (Labella et al., 2017; Cantos et al., 2019). The connection between *pipY* orthologs (PLPBP) and *proC*, encoding the metabolic enzyme pyrroline-5-carboxylate reductase, was previously suggested based on synteny (Prunetti et al., 2016). Synteny between the PLPBP coding genes from cyanobacteria (*pipY*), firmicutes, and actinobacteria (y*lmE*) with *sepF* (and other cell division genes) has been previously noted (discussed in Labella et al., 2017). However, the inferred functional connection between *engA* and *synpcc7942_2341* genes and of these with *pipX*, unlinked in the *S. elongatus* genome, has never been predicted by other methods. EngA is an essential GTPase involved in the maturation of the ribosomal subunit 50S (Hwang and Inouye, 2006; Bharat and Brown, 2014; Jeon et al., 2014). The predicted *synpcc7942_2341* gene product shows sequence similarity with bacterial EcfT (Energy Coupling Factor, T subunit) homologs, involved in micronutrient (vitamin or metal) import (Eitinger et al., 2011).

EngA (YphC/Der/YfgK), a protein conserved in bacteria, belongs to the GTPase superfamily (Hwang and Inouye, 2006; Bharat and Brown, 2014) and is composed of two GTPase-domains (GD1 and GD2) in tandem and a C-terminal domain resembling in its architecture a KH fold (RNA-binding module) (Hwang and Inouye, 2006; Verstraeten et al., 2011). In each of the GD domains, four characteristic sequence motifs (G1, G2, G3, and G4) (Bourne et al., 1991) are responsible for binding guanosine triphosphate (GTP) or guanosine diphosphate (GDP). These bindings are affecting interdomain interactions, switching the conformation of the whole protein between a closed state in which there is KH-GD1 interaction and an open conformation in which the KH domain is free from interactions with GD1. These changes ultimately modify EngA activity (Majumdar et al., 2017). Several structures of EngA corresponding to different conformations of this protein have been reported and analyzed (Robinson et al., 2002; Foucher et al., 2012; Zhang et al., 2014; Majumdar et al., 2017; Upendra and Krishnaveni, 2020), but none of these structures corresponds to EngA from cyanobacteria or from chloroplasts. Based on variations of the lengths of the GD1–GD2 linker and the KH-like domain, EngA proteins have been subdivided into f (firmicutes) and non-f groups (Majumdar et al., 2017), with cyanobacterial and gram-positive sequences included in the f group. The closest homologs of the plant chloroplast proteins (EngA1) are the cyanobacterial EngA proteins (Suwastika et al., 2014).

In this work, we expand the PipX interaction network, providing a proof of concept for the CLG. By two-hybrid and *in vitro* approaches, we demonstrate that PipX physically interacts with the GD1 domain of EngA. Genetic analyses in *S. elongatus* provided additional insights into the essential functions of cyanobacterial EngA and its regulatory connections with PipX. In the light of these analyses, we propose a model for the regulation of EngA by the hubs of the nitrogen interaction network.

## Materials and Methods

### Strains, Plasmids, and Oligonucleotides

The strains and plasmids are listed in Table 1. The oligonucleotides used in this work are listed in Table 2. The cloning procedures were carried out with *Escherichia coli* DH5α or XL1-Blue [for bacterial two-hybrid (BACTH) plasmids], using standard techniques (Sambrook et al., 1989). All constructs were analyzed by automated dideoxy DNA sequencing.

**TABLE 1:** Strains and plasmids.

Strain or plasmid	Genotype, phenotype, relevant characteristics	Source or references
*E. coli* DH5α	F^–^φ80 d*lacZ*ΔM15Δ(*lacZYA-argF*)U169 *endA1 recA1 hsdR17*(r_K_^–^ m_K_^+^) *deoR thi-1 supE44 gyrA96 relA1*λ^–^	Hanahan, 1985
*E. coli* BL21 (DE3)	F^–^ *ompT gal dcm lon hsdS*_*B*_(*r*_*B*_^–^*m*_*B*_^–^) λ(DE3 [*lacI lacUV5*-*T7p07 ind1 sam7 nin5*]) [*malB*^+^]_K–12_(λ^S^)	Studier et al., 1990
*E. coli* XL1-Blue	*recA1 endA1 gyrA96 thi-1 hsdR17 supE44 relA1 lac [F proAB lacIqZΔM15 Tn10 (Tet*^R^*)].*	Bullock et al., 1987
*E. coli* BTH101	F^–^, *cya-99*, *araD139*, *galE15*, *galK16, rpsL1*, *hsdR2*, *mcrA1*, *mcrB1*	Karimova et al., 2001
*Saccharomyces cerevisiae Y187*	MATα *ura3-52 his3-200 ade2-101 trp1-901 leu2-3, 112 gal4Δmet- gal80Δ URA::GAL1UAS-GAL1TATA-lacZ*	Harper et al., 1993
WT	Wild-type *Synechococcus elongatus* PCC7942	Pasteur Culture Collection
WT-Cm	Φ(NS2-*cat*), Cm^R^	This work
*pipX*	PipX^–^, Φ(P*_*pipX*_*::*cat*), Cm^R^	Labella et al., 2017
*engA^+/^* ^–^	EngA/EngA^–^, *engA/*Φ(P*_*engA*_*::*cat*), Cm^R^	This work
3^N^Ptrc	Φ(NS3-P*_*trc*_*), Nt^R^	This work
3^N^Ptrc-EngA	EngA^C^, Φ(NS3-P*_*trc*_*::*engA*), Nt^R^	This work
1^S^Ptrc-PipX	PipX^C^, Φ(NS1-P*_*trc*_*::*pipX*), Sm^R^	Labella et al., 2017
1^S^Ptrc-PipX/3^N^Ptrc-EngA	PipX^C^ and EngA^C^, Φ(NS1-P*_*trc*_*::*pipX*), Φ(NS3-P*_*trc*_*::*engA*), Sm^R^ Nt^R^	This work
3^N^Ptrc-EngA/*engA*^–^	EngA^C^, Φ(NS3-P*_*trc*_*::*engA*)/Φ(P*_*engA*_*::*cat*), Nt^R^ Cm^R^	This work
1^S^Ptrc-PipX/*engA^+/^*^–^	PipX^C^, EngA/EngA^–^, Φ(NS1-P*_*trc*_*::*pipX*), *engA/*Φ(P*_*engA*_*::*cat*), Sm^R^ Cm^R^	This work
pUT18c	CyaA(225–399)T18, Ap^R^	Karimova et al., 1998
pUT18	CyaA(225–399)T18, Ap^R^	Karimova et al., 2001
pKT25	CyaA(1–224)T25, Km^R^	Karimova et al., 2001
pKTN25	CyaA(1–224)T25, Km^R^	Karimova et al., 2005
pUAGC444	T18:PipX, Ap^R^	Espinosa et al., 2006
pUAGC934	PipX:T18, Ap^R^	This work
pUAGC1047	T25:PipX, Km^R^	This work
pUAGC1045	PipX:T25, Km^R^	This work
pUAGC920	T18:EngA, Ap^R^	This work
pUAGC1022	EngA:T18, Ap^R^	This work
pUAGC921	T25:EngA, Km^R^	This work
pUAGC1023	EngA:T25, Km^R^	This work
pUAGC1074	T18:NtcA, Ap^R^	This work
pUAGC1075	T25:NtcA, Km^R^	This work
pUAGC442	T18:PII, Ap^R^	Espinosa et al., 2006
pUAGC1048	T25:PII, Km^R^	This work
pUAGC1001	T25:PlmA	This work
pUAGC1024	T18:PipX^1–54^, Ap^R^	This work
pUAGC1026	T18:PipX^1–70^, Ap^R^	This work
pUAGC1096	PipX^1–54^:T18, Ap^R^	This work
pUAGC1095	PipX^1–70^:T18, Ap^R^	This work
pUAGC1025	T25:PipX^1–54^, Km^R^	This work
pUAGC1027	T25:PipX^1–70^, Km^R^	This work
pUAGC1103	PipX^1–54^:T25, Km^R^	This work
pUAGC1104	PipX^1–70^:T25, Km^R^	This work
pUAGC1070	T18:GD1, Ap^R^	This work
pUAGC1060	T18:GD1-GD2, Ap^R^	This work
pUAGC1064	T18:GD2-KH, Ap^R^	This work
pUAGC1072	GD1:T18, Ap^R^	This work
pUAGC1062	GD1-GD2:T18, Ap^R^	This work
pUAGC1066	GD2-KH:T18, Ap^R^	This work
pUAGC1071	T25:GD1, Km^R^	This work
pUAGC1061	T25:GD1-GD2, Km^R^	This work
pUAGC1065	T25:GD2-KH, Km^R^	This work
pUAGC1073	GD1:T25, Km^R^	This work
pUAGC1063	GD1-GD2:T25, Km^R^	This work
pUAGC1067	GD2-KH:T25, Km^R^	This work
pET-28a(+)	N-terminal 6xHis-tagged cloning vector with thrombin site	Novagen
pET-29c	N-terminal S-tag cloning vector with thrombin site	Novagen
pUAGC1039	6xhis-tagged EngA	This work
pUAGC1093	6xhis-tagged GD1	This work
pUAGC470	PipX–ST	Espinosa et al., 2006
pUAGC461	PipX–ST	This work
pUAGC126	PipX^–^, Ap^R^, Cm^R^	Labella et al., 2017
pGAD424	Amp^R^, *LEU2*, GAL4(768-881)AD	Bartel et al., 1993
pUAGC907	pGAD424 with *engAecfTC* genomic region, Ap^R^	This work
pUAGC908	EngA^–^, Ap^R^, Cm^R^	This work
pAM1580	*luxAB* into NS2, Ap^R^, Cm^R^	Andersson et al., 2000
pUAGC103	*P_*nblA*_::luxAB*, Cm^R^	Espinosa et al., 2006
pUAGC873	NS1, P_trc_::*pipX lacI*, Ap^R^, Sm^R^	Labella et al., 2017
pUAGC70	NS3, P_trc_ *lacI*, Nt^R^	Espinosa et al., 2018
pUAGC77	NS3, P_trc_::*engA lacI*, Nt^R^	This work

**TABLE 2:** Oligonucleotides.

Name	Sequence
pT25-seq	5′TCGGTGACCAGCGGC 3′
pUT18C-seq	5′GAAACGGTGCCGGCG 3′
pUT18-sec-F	5′TTCACACAGGAAACAGC 3′
pUT18-sec-R	5′GTCGATGCGTTCGCG 3′
pUT18c-sec-R	5′GGCTTAACTATGCGGC 3′
pKT25-sec-R	5′TGGGTAACGCCAGGG 3′
pKTN25-sec-R	5′ATGCCAGACTCCCGGTCG 3′
PipX–BTH-R54-2R	5′ATCCGGTACCGGACGACCGATTGGCTC 3′
PipX–BTH-R70-2R	5′GCAGGTACCGGTCGGCGCAGCTGGCG 3′
PipX–BTH-2F	5′CTCGGATCCCATGGCTTCCGAGAAC 3′
PipX–BTH-F	5′GATCGGGATCCCCGAGTAATG 3′
PipXresi70Rev	5′GCAGGAATTCCTATCGGCGCAGCTGGCGC 3′
PipXresi54Rev	5′ATCCGGAATTCCTAACGACCGATTGGC 3′
PipX–BTH-2R	5′CTCGGTACCGGCAGAAAGGTTTGTTTG 3′
2340-BTH-1F	5′CATGGATCCCATGCCGCTGCCGATT 3′
2340-BTH-1R	5′CATGGTACCCGAACGCGAGTCGCTCG 3′
EngA-BTH-2F	5′CATGGATCCCATCGGAGTCGCGATCG 3′
EngA-BTH-2R	5′CATGGTACCTTAGCGGCGACGGTGCTG 3′
EngA-BTH-4R	5′CATGGTACCTTAAACGCGAGTCGCTC 3′
GD1-BTH-1R	5′CATGGTACCTTATTCGGTTTCGTCCCCAGC 3′
GD1-BTH-2R	5′CATGGTACCCGTTCGGTTTCGTCCCCAGC 3′
EngA-BTH-355sin-R	5′CATGGTACCCGGCGGCGACGGTGCTG 3′
PII-BTH-F	5′GGAGGGATCCCTTGAAGAAG 3′
PII-BTH-R	5′TCGACGGTACCTTAGATTGCGTCG 3′
NtcA-BTH-2F	5′GGCATCTAGAGATGCTGGCCAACG 3′
NtcA-BTH-1R	5′TCCCTGGTACCGAGTGAGATTTAGC 3′
PlmA-BTH-1F	5′GCTGGATCCCATGATTCGTTTTCACATCC 3′
PlmA-BTH-1R	5′GCTGGTACCCGGTTCAAACCCAGTTCC 3′
PTRC99Aseq-F	5′GCCGACATCATAACGG 3′
7942NSIA-F	5′GACGGGTACCTCTGCTGG 3′
NSI-1F	5′CGACATCTTCCTGCTCCAG 3′
NSI-1R	5′TGCCTGAAAGCGTGACGAGC 3′
NSII-1F	5′AGGTTGTCCTTGGCGCAGCG 3′
NSII-1R	5′AGCGGATTTTGCATCACGAAGC 3′
NS3-seq-1F	5′ACCTCCGGCAGTCAATTA 3′
NS3-seq-1R	5′AGGGACTGGTTGATCGGT 3′
2340-For	5′CCGAGGATCCTGATGTGACTGGCGC 3′
2341-rev	5′CAGAGTCGACGCCATTGACTGAGG 3′
2340-Cm-1F	5′ATGACTAGAATGAAGGTTTGCATGTAAGTAAGTTGTTAT GGAGAAAAAAATCACTGGATA 3′
2340-Cm-1R	5′ACCCCAACGGCAGCGATCGCAGTAAATCCATTGCAGC AGTTTACGCCCCGCCCTGCCAC 3′
2340-OV-1F	5′AAGTAAGAGCTCATGCCGCTGCCGA 3′
2340-OV-1R	5′TTGCTCTAGATTAAACGCGAGTCGC 3′
GD1-EngA-Rev	5′CCCCTGCAGTTAATCCGCCGGGGGCAATAG 3′
PIPX-pTRC-1F	5′GGTTCCCATGGGACACCATCACCATCACCATATGGCTT CCGAG 3′
Ptet::pipX-1R	5′TTCACAGATCTAGCTTATTATTTTTCG 3′

### Plasmid Construction

The construction details of the BACTH plasmids (primers and enzymes used, cloning vector, and final fusion protein expressed) are given in Supplementary Table 1. In brief, the relevant coding region was PCR-amplified, digested with two enzymes, and cloned into the corresponding sites of BACTH vectors.

To construct pUAGC907, the genomic region containing *engA* and the downstream gene *Synpcc7942_2341* were PCR-amplified with primers 2340-For/2341-Rev, digested with *BamH*I and *Sal*I, and cloned into pGAD424. pUAGC907 linearized with *BamH*I was used in combination with PCR-amplified (using primer pair 2340-Cm-1F/1R) *cat* sequences from pUAGC103 to obtain plasmid pUAGC908 by homologous recombination in yeast (using strain Y187). To obtain pUAGC77, *engA* genomic sequences were PCR-amplified using the primers 2340-OV-1F/1R and then cut with *Ecl136*II/*Xba*I and cloned into pUAGC70.

To construct pUAGC1039, a fragment comprising the complete *engA* ORF was excised from pUAGC77 with *Ecl*136II and *Xho*I and cloned into pET-28a(+). To obtain pUAGC1093, an *engA* fragment corresponding to the GD1 domain (1–169 amino acids) was PCR-amplified with 2340-OV-1F/GD1-EngA-Rev. The resulting 531-bp product was digested with *Ecl136*II and *Pst*I and cloned into pUAGC1039.

To construct pUAGC461, the *pipX* strep-tag fusion sequence was PCR-amplified from pUAGC470 using primers PIPX-pTRC-1F/P*tet::pipX*-1R to generate a 348-bp product that was cut with *Nde*I and *Bgl*II and cloned into pET-29c.

### Cyanobacteria Culture Conditions, Strain Construction, and Growth Determination

*S. elongatus* strains were routinely grown photoautotrophically at 30°C while shaking under constant illumination (40 μmol photons m^–2^s^–1^) provided by cool white fluorescent lights. The medium used was blue-green algae medium BG11 (BG11_0_ plus 17.5 mM NaNO_3_ and 10 mM HEPES/NaOH, pH 7.8). For growth on plates, the media was solidified by the addition of 1.5% (w/v) agar. The plates were routinely incubated at 30°C under constant illumination. *S. elongatus* strains were transformed essentially as described by Golden and Sherman (1984).

To inactivate *engA*, *S. elongatus* was transformed with pUAGC908. The transformants were selected with chloramphenicol (2.5 μg ml^–1^) and subsequently analyzed by PCR with oligonucleotides 2340-For/2341-rev to distinguish the wild-type (2,713 bp) and mutant (2,011 bp) alleles. To generate the strain WT-Cm, *S. elongatus* was transformed with plasmid pAM1580, and the transformants were verified by PCR with primers NSII-1F/NSII-1R.

To overexpress *engA* in *S. elongatus*, pUAGC77 transformants were selected with nourseothricin (10 μg ml^–1^) and verified by PCR with the primer pairs PTRC99Aseq-F/GD1-BTH-R and NS3-seq-1F/NS3-seq-1R.

To overexpress *pipX* in *S. elongatus*, pUAGC873 transformants were selected with streptomycin (2.5 μg ml^–1^) and verified by PCR with primer pairs PTRC99Aseq-F/NSI-1F and 7942NSIA-F/NSI-1R.

To determine the growth rate in liquid cultures, the following formula was used:


$$G\,r\,o\,w\,t\,h\,r\,a\,t\,e\,\left(h^{-1}\right)=\frac{l\,n\,\frac{O\,D_{f}}{O\,D_{i}}}{24}$$

where *OD_*i*_* corresponds to the initial optical density adjusted every 24 h (∼0.1), and *OD*_*f*_ is the optical density after 24 h of growth. Cultures (from three independent clones) were grown for an additional 48 h, adjusted to 0.1 (OD_750_), and then supplemented with 1 mM IPTG. Every 24 h, the cell absorbance was readjusted to 0.1 with fresh medium (BG11 plus 1 mM IPTG).

For the quantitative estimation of growth on solid media, exponentially growing cultures (mix of three clones) were adjusted to 0.5 (OD_750_) before plating 5 μL of cell suspensions and the corresponding dilutions (5^–1^, 10^–1^, and 10^–2^). Plates were grown under constant illumination at 30°C (4 days) or 20°C (14 days) before photographs were taken using a Nikon camera at the default parameters. The photographs were analyzed using ImageJ. Circular regions of interest (ROIs) were manually generated for each drop, and the average pixel intensity in the red channel was retrieved. An empty ROI was generated to obtain the background noise value of each picture which was subtracted from the other ROI average values. The relative growth of each strain relative to the control strain was calculated as the minimum value of the ratios between the measured biomass values of drops of the same dilution.

To compare the ability of the different strains to resume growth after the stress treatment, cultures were adjusted to 0.1 (OD_750_) before 5 μL were spotted on BG11 plates. Photographs were taken after 72 h of incubation under standard conditions.

### Cytometry Measurements

Cytometry measurements were performed with a BD FACSCanto Analyzer (FACSDiva v.8 software). To remove non-cellular events, cytometry measurements were filtered using the criteria APC-A (633 nm laser) > 100 and PerCP-Cy5-5-A (488 nm laser) > 80. All values were normalized using the FSC-A value, and the resulting extreme values (higher than the third quartile plus the interquartile distance or lower than the first quartile minus the interquartile distance) were removed. Finally, the control strain average value was used to normalize the other strain values within experiments.

### Microscopy, Image Acquisition, and Analysis

Exponentially growing cells (5 μL) were mounted on 2% low-melting-point agarose pads for microscopy. To image cell auto-fluorescence and vancomycin staining (BODIPY™ FL vancomycin; final concentration 1 μg ml^–1^, 20 min 37°C), a TCS-SP2 Leica confocal microscope was used as described in Labella et al. (2017) (running under Leica Confocal Software version 2.61, Leica Microsystems). The filter specificities were as follows: ex633 nm/em665–700 nm (for cyanobacterial auto-fluorescence analysis) and ex488 nm/em495–537 nm (for vancomycin).

To visualize DNA compaction, cells were stained with 4′,6-diamidino-2-phenylindole (DAPI) at 8.9 μg ml^–1^ for 10 min at 30°C. Micrographs were taken using a confocal laser scanning microscope (Zeiss model LSM800). The filter parameters were as follows: ex640 nm/em650+ nm (for cyanobacterial auto-fluorescence analysis) and ex405 nm/em410–470 nm (for DAPI).

### BACTH Assays

*E. coli* BTH101 was co-transformed with appropriate plasmids and colonies were selected on ampicillin (50 μg ml^–1^) and kanamycin (40 μg ml^–1^) LB plates. Five clones from each plate were inoculated into 0.5 ml of LB plus antibiotics plus 0.5 mM IPTG and incubated at 30°C for 24 h. Interactions were assayed by dropping 3 μL of each saturated culture on M63 (containing 0.3% maltose, 0.0001% thiamin, 1 mM magnesium sulfate, 0.5 mM IPTG, and 40 μg ml^–1^ X-gal) and MacConkey (containing 1% lactose and 0.5 mM IPTG) reporter plates and incubated for 48 h (MacConkey) or 72 h (M63) at 30°C. Photographs were taken at 24-h intervals.

### Protein Purification in *E. coli*

His-EngA, His-GD1, and PipX–ST proteins were overexpressed and purified from *E. coli* BL21-DE3. Strains carrying pUAGC1039, pUAGC1093, or pUAGC461 were grown to 0.6–0.8 (OD_600_) before the addition of 0.5 mM IPTG and incubated overnight at 16°C. Cells were harvested by centrifugation (8,000 *g* for 10 min), resuspended in a lysis buffer (50 mM Tris-HCl, pH 8, 250 mM NaCl, 1 mM DTT, and 1 mM PMSF), and sonicated through 10 1-min rounds on ice, with a 2-min break between each round), with output = 10 and 50% duty cycle (Branson Sonifier 250). The lysate was then centrifuged for 10 min at 30,000 *g* and 4°C to remove cell debris, and the supernatant was centrifuged for 20 min with the same settings. To purify His-EngA and His-GD1 proteins, the corresponding supernatant was applied onto a Ni-Sepharose prepacked column (His GraviTrap, Cytiva) previously equilibrated, subsequently washed, and finally eluted following the recommendations of the manufacturer (Supplementary Figure 1). Elution fractions were first analyzed by SDS-PAGE and Coomassie staining and then pooled and diafiltrated in storage buffer (50 mM Tris-HCl pH 8, 500 mM NaCl, 1 mM DTT, and 10% glycerol) using Amicon Ultra-4 Centrifugal Filter Devices. The absorbance spectra of His-EngA proteins yield a Abs_260/280__nm_ ratio of about 0.7, indicative of no contamination with nucleic acids (Supplementary Figure 1). To purify PipX–ST, cell-free supernatant was loaded onto Strep-Tactin XT 4Flow columns, and purification was carried out following the recommendations of the manufacturer. Elution fractions enriched with PipX–ST were pooled and diafiltrated in storage buffer (50 mM Mes-NaOH, pH 6, 50 mM L-Arg, 50 mM L-Glu, 500 mM NaCl, 1 mM DTT, and 10% glycerol). A colorimetric method, based on the Biorad DC protein assay reagent, was used to determine His-EngA, His-GD1, and PipX–ST protein concentrations.

### Pull-Down Assays

*In vitro* protein complex formation was assessed using DynaBeads-Histag (Invitrogen). All reactions were conducted in binding buffer containing 50 mM Tris-HCl, pH 7, 0.1 M KCl, 10% glycerol, 10 mM MgCl_2_, 20 mM imidazole, and 0.1% Triton-X100 in the presence or absence of 1 mM GDP. Then, 7 μL of beads and 6 μg of His-EngA or His-GD1 per assay were equilibrated in binding buffer. After the initial binding of EngA to Dynabeads (10 min at room temperature, RT), 50 μL of binding buffer, including 6 μg of purified PipX–ST and supplemented or not with 1 mM GDP, was added. A control sample lacking EngA and containing only beads and 6 μg of purified PipX–ST was run in parallel. After 10 min at RT, the beads were washed four times (4 min/wash) with 300 μL of buffer supplemented or not with GDP. A final 25-μL wash was recovered, prior to elution, together with the 25-μL elution fraction obtained after incubation with the buffer supplemented with 500 mM imidazole.

### Immunodetection of PipX and EngA by Western Blotting

Rabbit antisera against EngA were obtained from Pineda Antikörper Service (Berlin, Germany) using ∼2 mg of pure recombinant His-EngA as antigen and following a 60-day immunization protocol. The antiserum against PipX protein was donated by K. Forchhammer (Univ. Tübingen, Germany). For immunodetection of PipX and EngA proteins from pull-down fractions, 8 μL (one-third of the final fraction volume) was subjected to SDS-PAGE (10–20% polyacrylamide linear gradient), followed by immunoblotting using 0.1-μm polyvinylidene fluoride membranes (from GE Healthcare). For immunodetection of EngA in *S. elongatus* protein extracts, cells from liquid cultures were harvested at different times after addition of 1 mM IPTG and subsequently lysed as described in Labella et al. (2016) using glass beads and a cell disruptor (three pulses of 1 min separated by 1-min ice-cooling intervals). PipX and EngA antisera were diluted at 1:5,000, and the secondary anti-rabbit-HRP antibody was used at 1:150,000 dilution. Visualization was performed with SuperSignal WestFemto reagent (Pierce) and the signal recorded in a Biorad ChemiDoc Imager using the automatic exposure mode and avoiding pixel saturation.

## Results and Discussion

### Bacterial Two-Hybrid Analyses of Interactions Mediated by PipX, Nitrogen Regulators, and EngA

**FIGURE 2:**
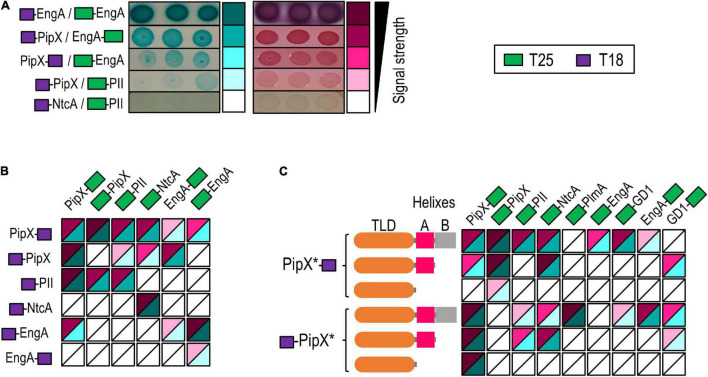
Two hybrid analyses of PipX interactions with nitrogen regulators and EngA. **(A)** Examples of *Escherichia coli* BTH101 co-transformants expressing the indicated fusion proteins on plate assays, ordered according to the intensity of the interaction signals and classed into five levels. The relative position of the CyaA domains (T25 and T18; see the inset for color code) is indicated in each case. Pictures were taken after 24 h (MacConkey–lactose) and 72 h (M63-maltose + X-gal) of incubation at 30°C. Heat maps integrating the results from **(B)** PipX, EngA, PII, and NtcA in “all against all” assays or **(C)** PipX and derivatives (represented as PipX* and schematically illustrated to the left) against the indicated proteins (see Supplementary Figure 2 for representative examples of the corresponding plate assays).

In the context of exploring functional connections within the PipX synteny network, we investigated whether PipX and EngA can bind to each other by using the BACTH system. This genetic approach to protein–protein interactions is based on the reconstitution of the adenylate cyclase of *Bordetella pertussis* in *E. coli* (Karimova et al., 1998) and produces virtually no false positives (Battesti and Bouveret, 2012). To discriminate among different levels of interaction signals with reasonable confidence on plate assays, these included three independent pools of five clones (co-transformants for the appropriated pairs) that were tested in parallel on MacConkey + lactose and on M63 + maltose + X-gal (Supplementary Figure 2). In this way, we obtained reproducible information for two different reporters (*lac* and *gal*) as well as from two different environmental conditions (rich and non-selective vs. minimal and selective media). By visual observation of the intensity of the red and blue colors, we classified signals obtained from the different assays into five categories ranging from “no interaction” to “very strong” (Figure 2A). The resulting heat map scale is used hereafter to represent results from additional BACTH assays in a friendly and integrative manner and to facilitate awareness of possible discrepancies between the two growth conditions used for plate assays as well as of the common occurrence of false negatives. Note that while the strength of the signal is not a direct measure of protein affinity, ranking the different levels of signals does allow a comparison of protein variants within a given pair of interacting proteins.

To provide internal controls for the specificity and reliability of the assays, as well as to explore the possibilities of using the BACTH system to map domain determinants within the PipX interaction regulatory network, we focused on PipX, its known binding partners PII and NtcA, and the query (EngA). To this end, we tested the N-terminal fusions of each of the T18 and T25 domains to PipX, PII, and NtcA as well as the C-terminal fusions of T18 and T25 to PipX and EngA, giving a total of 12 constructs. This allowed eight cross-interaction tests for PipX/EngA, four for each of these proteins against PII or NtcA and two for the internal negative control (PII-NtcA) as well as self-interaction assays (four in the cases of PipX and EngA) (Figure 2B). Self-interaction, expected for proteins with quaternary structure such as PII (trimer) and NtcA (dimer), provides positive controls for the correct design and functionality of the cloned proteins, as it is the case for the T18-NtcA construct (failing to interact with PipX derivatives in our assays). Interestingly, we obtained self-interaction signals with all four proteins: PII/PII (1/1), NtcA/NtcA (1/1) PipX/PipX (3/4), and EngA/EngA (3/4).

Previously, we detected PipX self-interaction in yeast three-hybrid assays using PII (not homologous in yeast) as bridge, but not in classical two-hybrid assays, in line with the fact that PipX forms stable trimers when bound to *S. elongatus* PII (Llácer et al., 2010). Our interpretation of the PipX self-interaction found in BACTH assays is that the presence in the *E. coli* host of PII proteins GlnB and GlnK may provide the binding partner(s) required for PipX. Therefore, this may be illustrative of the ability of the BACTH system to detect indirect interactions, presumably helped by *E. coli* proteins acting as bridges between the proteins fused to the T18 and T25 domains (Battesti and Bouveret, 2012). In the case of EngA, self-interaction may reflect its ability to form dimers or higher-order structures *in vivo*, perhaps aided by effector molecules that would be lost during purification procedures. In line with this, a self-interaction of *E. coli* EngA has also been reported in a yeast two-hybrid system (Hwang and Inouye, 2010).

Robust interaction signals were found for PipX/PII (all 4) and PipX/NtcA (2/4) pairs (Figure 2B) but not for the PII/NtcA negative controls (0/2), thus indicating that the BACTH system reflects the specific interactions taking place within components of the nitrogen signaling system. Importantly, positive results with relatively strong and reproducible signals were obtained for PipX/EngA pairs (4/8), supporting the functional relevance of the PipX synteny network and prompting us to use the BACTH system to investigate the binding determinants involved.

### Interactions With EngA Involves Structural Elements Outside the TLD/KOW Domain of PipX

In previous studies, we tested yeast two-hybrid fusions of truncated PipX polypeptides PipX^1–70^ (lacking helix B) and PipX^1–54^ (lacking helices A and B) against PII, and only the largest one (PipX^1–70^) gave interaction signals (Labella et al., 2016). To investigate the PipX determinants for interaction with EngA and get additional information on the interactions mediated by the PipX^1–70^ and PipX^1–54^ fragments in two-hybrid assays, we now constructed N- and C-terminal fusions of each of the T18 and T25 domains to each of the truncated derivatives of PipX and tested them against N-ter and C-ter fusions of T25 to EngA or control proteins interacting with PipX. PlmA, a protein that requires the presence of PII to interact with PipX in the yeast system (Labella et al., 2016), was also included in the assays to explore the possibility of *E. coli* PII proteins acting as bridge, as suggested by the PipX self-interaction results. The results of all pairwise interactions are summarized in Figure 2C.

Interaction signals were found for one of the two PipX/PlmA pairs, a result supporting the idea that GlnB and/or GlnK proteins may be binding to PipX and subsequently facilitating contacts with PlmA. In addition, the removal of helix B impaired the interaction signals with PlmA, a result which is in close agreement with previous yeast three-hybrid assays (Labella et al., 2016). While PipX and PipX^1–70^ gave signals with NtcA and PII, none of the PipX^1–54^/NtcA or PipX^1–54^/PII pairs did. However, self-interaction signals were found for each of the truncated derivatives paired with PipX, thus indicating that the TLD/KOW domain is responsible for PipX self-interaction but that, apparently, it is not sufficient to give BACTH signals with NtcA or PII in the absence of helix A residues.

Importantly, none of the PipX^1–54^ or PipX^1–70^ derivatives gave signals with EngA (0/3 informative PipX/EngA pairs analyzed), indicating that at least helix B was required for interaction with full-length EngA. Therefore, the mode of binding of PipX to EngA must differ from that involving the PipX–PII and PipX–NtcA paradigms.

### The GD1 Domain Contains the Main Determinants for Binding to PipX

**FIGURE 3:**
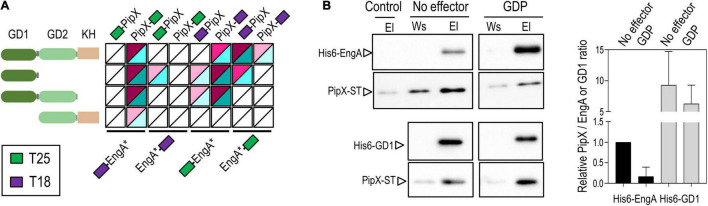
PipX binds to the GD1 domain of EngA. **(A)** Heat maps integrating BACTH analysis involving PipX and EngA and its derivatives (represented as EngA*). The other details are as in Figure 2. **(B)** Representative immunodetection blot showing the interaction between either purified His-EngA or His-GD1 and PipX–ST proteins assessed by pull-down in the absence or presence of 1 mM guanosine diphosphate. From each pull-down, the elution (El) and the previous wash (Ws) were analyzed by Western blot using specific antibodies raised against PipX and EngA. The panels correspond to pieces of the same immunoblot loaded with the pull-down samples using His-EngA (top panels) or His-GD1 (bottom panels). The intensity of each PipX band in the eluates, from at least two independent pull-downs for each condition, was determined and corrected by subtraction using the eluate of a pull-down omitting EngA (control lane). The bars from the histogram show the PipX/EngA or PipX/GD1 band intensity ratio in each condition from two independent experiments relative to the PipX/EngA ratio in the absence of effectors (set arbitrarily to 1).

To map the determinants of EngA involved in binding to PipX, we obtained truncated derivatives lacking the N- or C-terminal domains (GD2-KH or GD1–GD2 constructs, respectively) or just expressing the N-terminal domain (GD1) of all four types of BACTH constructs (N- and C-terminal fusions of each of the T18 and T25 domains to the different polypeptides) and tested them against the corresponding PipX fusions in all possible combinations (Figure 3A). The EngA polypeptide with more constructs interacting with PipX was GD1, followed by EngA and GD1–GD2. Importantly, the deletion of GD1 (GD2-KH constructs) either abolished or greatly impaired the interaction signals, indicating that GD1 contains the main determinants for PipX binding. The weak interaction signals provided by the PipX–T25/T18-GD2-KH pair also suggested that GD2 could contain a low-affinity binding site for PipX that may function as such in the absence of GD1 and when the GD2-containing polypeptide is overproduced relative to PipX (T18 fusion proteins are expressed from multicopy vectors).

To study whether domains not providing the interaction surfaces at PipX–EngA complexes may play regulatory roles, we performed additional BACTH analysis to test if the C-terminal helices of PipX were still required when the GD1 becomes more accessible, that is, in the absence of the GD2-KH domains. Interaction signals between PipX^1–70^ and GD1 were obtained with two different fusion pairs (Figure 2C), indicating that, instead of being required to maintain PipX–EngA contacts, the positive role of helix B on the interaction with EngA may be regulatory. The implication is that the main determinants for interactions with the GD1 domain of EngA could be at the TLD/KOW domain of PipX.

Taken together, the BACTH analyses indicate that domains of EngA as well as T18 and T25 domains affected the balance of EngA conformations within the different fusion proteins, exposing or occluding determinants for interactions with PipX. The results also suggest that PipX uses its C-terminal region to facilitate contacts with EngA, thus calling attention to the regulatory complexity and multiplicity of factors that may affect the encounters of the two proteins in cyanobacterial cells.

The identification of GD1 as the main EngA determinant for PipX binding contrasts with the case of the *E. coli* EngA (Der) and YihI, the only modulator of an EngA protein identified so far (Hwang and Inouye, 2010). YihI is a highly hydrophilic protein, with no resemblance in protein sequence or structure with PipX that physically interacts with the GD2 and KH domains of Der. The mutually exclusive phylogenetic distribution of PipX and YihI (YihI is absent from cyanobacteria) fuels speculation on the possible existence of additional and novel EngA binding proteins awaiting discovery in gram-positive bacteria, which do not encode PipX or YihI proteins.

### Pull-Down Assays With Purified Proteins Confirm the Direct Binding of EngA to PipX

To corroborate the binding of PipX to EngA and to the GD1 domain of EngA *in vitro*, we used cell-free pull-down assays with His-tag magnetic beads, for which we obtained C-terminally Strep-tagged PipX (PipX–ST) and N-terminally His-tagged EngA (His-EngA) or its GD1 derivative (His-GD1). Proteins were detected after Western blotting of the corresponding pull-down fractions, using specific antibodies raised against PipX or EngA, and the intensities of the immunodetected bands were used to determine the relative quantity of each protein in each fraction. In previous tests with our custom Anti-EngA, we found that identical known amounts in the mass of His-EngA and His-GD1, corresponding to a 1:2.5 molar ratio (EngA:GD1), produced similar band intensities (Supplementary Figure 3). The pull-down control detected a low binding of PipX–ST to the beads that clearly increased when His-EngA or His-GD1 were included in the assays, thus indicating the specific retention of PipX–ST by binding His-EngA or His-GD1 (Figure 3B). Importantly, the *in vitro* experiment also shows that no PII or additional proteins are required for PipX–EngA complex formation.

To compare the levels of retention of PipX–ST by His-EngA and His-GD1, the intensities of each PipX band were normalized by that of the corresponding EngA or GD1 bands from the same elution fraction (Figure 3B, left panel). The intensities of the immunodetected EngA and GD1 protein bands were very similar when the elution fractions of different pull-down experiments were analyzed, indicating a retention of 2.5 times more molecules of His-GD1 than of His-EngA. The intensity of the immunodetected PipX and EngA or GD1 bands was determined and used to calculate the PipX/EngA or PipX/GD1 ratio as an indicator of the amount of PipX recovered by the amount of either EngA or GD1. The PipX/EngA ratio from the pull-down condition carried out with His-EngA was used as a reference to normalize and set arbitrarily to 1. Ratio comparisons showed that the retention of PipX was approximately one order of magnitude higher with GD1 than with EngA, in complete agreement with our BACTH results indicating that GD1 contains the main binding determinants.

Next, we tested the influence of GDP, known to cause conformational changes in EngA orthologs (Muench et al., 2006). When the PipX/EngA ratios in the presence of GDP were compared with those obtained in its absence, we found a significant decrease in the amount of immunoprecipitated PipX, thus suggesting that GDP drives EngA from a conformation favorable to PipX binding to an unfavorable one. In this context, it is tempting to propose that the *Bacillus subtilis* GDP-bound structures determined for EngA (2HJG PDB structure), corresponding to a closed conformation, would be one of the EngA conformations not binding to PipX *in vivo*.

### EngA Is Essential in *S. elongatus* and Its Depletion Has Pleiotropic Effects

EngA plays essential roles in ribosome biogenesis in both bacteria and chloroplasts (Hwang and Inouye, 2006; Bharat and Brown, 2014; Jeon et al., 2014) and has been found to be associated with membranes in *E. coli* (Lee et al., 2011) and with thylakoids, where it affected the photosystem II repair cycle, in *Arabidopsis thaliana* (Kato et al., 2018). Several phenotypes have been associated with the depletion of EngA, including cold sensitivity, cell filamentation, abnormal cell curvature, apparent nucleoid condensation, and, in plants, aberrant thylakoid organization (Hwang and Inouye, 2001; Morimoto et al., 2002; Bharat and Brown, 2014; Jeon et al., 2014). So far, *engA* has proven essential in all systems studied (Mehr et al., 2000; Hwang and Inouye, 2001; Morimoto et al., 2002; Zalacain et al., 2003) and, not surprisingly, is also listed in the essential gene set of *S. elongatus* (Rubin et al., 2015).

**FIGURE 4:**
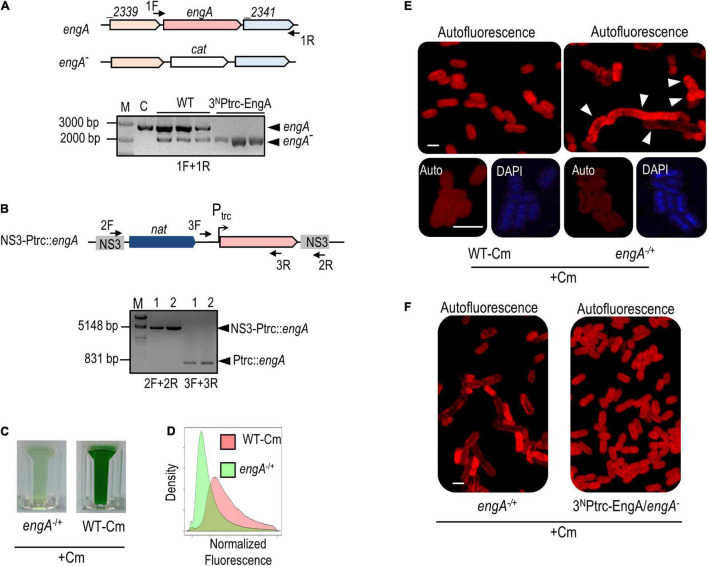
*engA* is an essential gene in *Synechococcus elongatus*, and its depletion is pleiotropic. **(A)** Schematic representation of wild-type and *engA* inactivation alleles followed by PCR analyses showing the incomplete or complete segregation of the *engA* null allele in wild type (WT) and 3^N^Ptrc-EngA backgrounds, respectively. **(B)** Schematic representation of the allele used for the ectopic expression of *engA* and PCR verification of its correct structure in two independent clones. For **(A,B)**, the position of the PCR primers is shown by arrows in the schemes; amplified alleles or chromosomal regions (arrowheads) and reference size bands (numbers) are indicated, respectively, to the right and the left of PCR gels; M, marker; C, control strain. **(C)** Visual appearance of cultures of *engA*^–/+^ and WT-Cm strains 3 days after being inoculated with the same amount of biomass. **(D)** Density plot of the autofluorescence signal of cells by flow cytometry, normalized by cell size (FSC-A, ex633 nm) and control signal. **(E,F)** Autofluorescence and **(E)** DAPI signals of indicated strains grown until 0.5–0.8 at OD_750_. Scale bar, 3 μm. The arrowheads in **(E)** point out the morphologically affected cells.

To confirm the essentiality of *engA* and get insights into the function of cyanobacterial EngA protein, we attempted to inactivate the *engA* gene in *S. elongatus* by allelic replacement, precisely substituting the coding region by that of the selectable *cat* gene, encoding chloramphenicol-acetyltransferase (Figure 4A). Due to the presence of multiple chromosome copies in cyanobacteria (Watanabe, 2020), gene inactivation in *S. elongatus* requires verification that allelic replacement has been completed. In this context, although chloramphenicol-resistant (Cm^r^) clones could be obtained when *S. elongatus* was transformed with the inactivation constructs, all analyzed clones retained the WT *engA* allele (Figure 4A), indicating that *engA* is required for the survival of *S. elongatus*. To detect possible polar effects over the downstream ORF, we next asked whether the ectopic expression of *engA* rescued the null mutant by inactivating *engA* in a *S. elongatus* strain ectopically expressing a wild-type version of this gene, constructed with this purpose (3^N^Ptrc-EngA, Figure 4B). As expected, segregation of the Cm^r^ transformants was readily achieved in the 3^N^Ptrc-EngA background (Figure 4A), thus excluding polar effects while validating the strategy for the inactivation of *engA*, clearly an essential gene in *S. elongatus*.

Heterozygote *engA*^–/+^ clones grew very slowly in the presence of Cm (Figure 4C), offering limited opportunity for analysis before they died. It is worth noting that, because of random segregation of three to four chromosomal copies of *S. elongatus* (Watanabe, 2020), heterozygotic cultures tend to be very heterogeneous and unstable, adding variability to experimental results. To identify features associated with EngA depletion, we first compared the morphological appearance of *engA*^–/+^ cells with those of a control strain obtained by inserting the *cat* gene into a neutral site (NS2) of the *S. elongatus* chromosome (WT-Cm). Flow cytometry showed that the cell auto-fluorescence was lower in comparison to control cells (Figure 4D). Confocal microscopy of *engA*^–/+^ cultures revealed morphologically very heterogeneous cells, with abnormal distribution of the auto-fluorescence signal, indicative of aberrant thylakoid organization. A significant proportion of the cells was abnormally large, long, and/or curved, while the normal-looking cells showed higher DAPI fluorescence from nucleoids, suggestive of chromosome condensation (Figure 4E). In agreement with complementation results, cells from 3^N^PtrcEngA/*engA*^–^ showed a wild type phenotype under the microscope (Figure 4F).

The pleiotropic phenotype and some of the features of EngA-depleted cultures are in line with the phenotypic effects reported (often separately) in other systems. Regarding the abnormal appearance and phenotypic diversity of mutant cells, there are very few studies reporting the effects on cell appearance of lethal (either null or gain-of-function) mutations in *S. elongatus* or in other cyanobacteria, so we are uncertain of whether these changes would also occur in other translation-related mutants of cyanobacteria or are rather specific of EngA-depleted cells.

### Increasing the Levels of EngA Appears to Have No Phenotypic Consequences

EngA overexpression in *E. coli* induces changes in cell wall structure and morphology, with clear spaces or holes at the cell poles (Lee et al., 2011), while in *A. thaliana* the overexpression phenotypes include leaf variegation, high chlorophyll fluorescence, increased ROS generation, and compromised PSII repair (Kato et al., 2018). To investigate the phenotype of overexpression of EngA in *S. elongatus*, we analyzed the effect of IPTG addition in 3^N^Ptrc-EngA and 3^N^Ptrc strains, which just differed in the presence of *engA* sequences at the NS3 chromosomal site.

**FIGURE 5:**
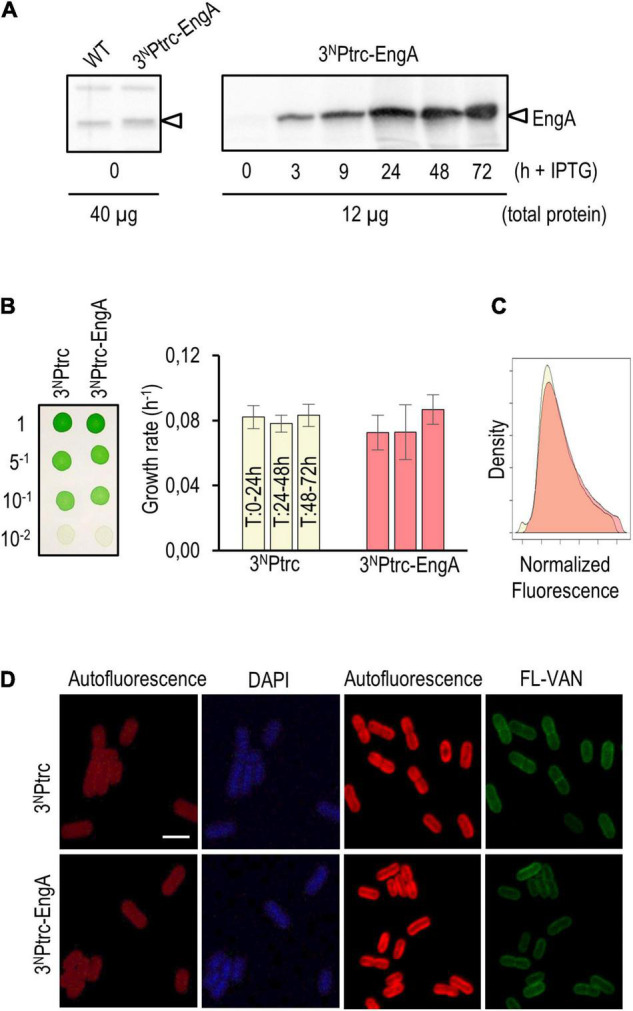
Phenotypic effects of EngA overexpression in *Synechococcus elongatus*. **(A)** Immunodetection of EngA (indicated with an arrowhead) in wild-type and 3^N^Ptrc-EngA strains (left panel) and time course induction after the addition of 1 mM IPTG to strain 3^N^Ptrc-EngA (right panel). **(B)** Growth tests on solid (left) and liquid (right) media plus 1 mM IPTG. **(C)** Density plot of the autofluorescence signal of cells measured by flow cytometry, normalized by cell size (FSC-A, ex 633 nm) and control signal. **(D)** Cells grown for 72 h (DAPI) or 24 h (FL-VAN) with 1 mM IPTG were visualized under a confocal microscope. Scale bar, 3 μm. For **(B–D)**, three independent clones were previously mixed.

Western blots confirmed that strain 3^N^Ptrc-EngA accumulates EngA protein in an IPTG-dependent manner. The levels of EngA in 3^N^Ptrc-EngA were just slightly higher than those of the control (Figure 5A, left panel) and significantly increased after IPTG addition, remaining very high during the 24–72-h interval analyzed (Figure 5A, right panel). As shown in Figure 5B, no growth differences were found between 3^N^Ptrc-EngA and control strain 3^N^Ptrc, neither in solid nor in liquid cultures with IPTG. The flow cytometry patterns and cell appearance regarding auto-fluorescence, DAPI, and FL-vancomycin signals were the same for the 3^N^Ptrc-EngA and 3^N^Ptrc strains (Figures 5C,D). Thus, the neutral phenotype of EngA overexpression contrasts with results reported in other model systems, raising the questions on the possible regulatory differences distinguishing cyanobacteria from those other systems.

### Gene Interactions Between *pipX* and *engA* in *S. elongatus* Support the Importance of Maintaining a Low PipX/EngA Ratio

The inactivation of *pipX* has no apparent effects on growth (Espinosa et al., 2009; Labella et al., 2017). However, *pipX* overexpression or gain-of-function mutations interfere with *S. elongatus* growth, a “PipX toxicity” phenomenon observed whenever the PipX/PII ratio is increased (Espinosa et al., 2009, 2010; Laichoubi et al., 2012; Chang et al., 2013; Labella et al., 2020a; Sakamoto et al., 2021). In this scenario, we have been speculating that PipX toxicity could be caused by its binding to an unknown and low-affinity binding partner, and we now wondered whether EngA could be the enigmatic PipX partner.

**FIGURE 6:**
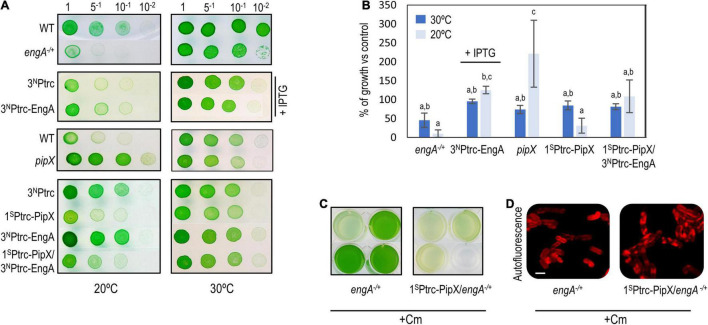
Gene interactions between *pipX* and *engA* in *Synechococcus elongatus*. **(A)** Representative growth tests (from at least three experiments) of the indicated strains and temperature conditions. **(B)** Average and standard deviation of growth percentage (as ratio of biomass) of the indicated strains compared to their corresponding controls [wild type for *engA*^+/–^ and *pipX* (*pipX* null mutant); 3^N^Ptrc for 3^N^Ptrc-EngA, 1^S^Ptrc-PipX, and 1^S^Ptrc-PipX/3^N^Ptrc-EngA] at 30 or 20°C. Biomass from pictures was measured using Image J. Bars are grouped into three overlapping categories labeled as a, b, and/or c, according to the results of a pairwise *t*-test with Bonferroni correction. Bars sharing at least one common label do not show statistical differences (*p*-value < 0.01). For **(A,B)**, IPTG was added only to the indicated cultures. **(C)** Visual appearance of the indicated Cm-resistant cultures 10 days after being inoculated with the same amount of biomass. **(D)** Cell morphology and autofluorescence levels in confocal micrographs (ex633 nm); scale bar, 3 μm.

To explore the possible implication of EngA in the phenomenon of PipX toxicity, we performed an additional genetic analysis. Since alteration of *pipX* and *engA* gene dosages can have very severe effects on culture viability, the genetic analysis is not straightforward due to factors such as cell heterogeneity and/or the convenience of using or not selective (Cm) or induction (IPTG) conditions in each case. This also limits the panel of *S. elongatus* strains with significant changes in the levels of EngA and PipX proteins that can be tested without compromising the interpretation of results due to excessive lethality. Therefore, the strains analyzed in this context included *engA*^–/+^, *pipX* null, and strains ectopically expressing one or both proteins: 1^S^Ptrc-PipX, 3^N^Ptrc-EngA (in the presence of the IPTG inducer), and 1^S^Ptrc-PipX/3^N^Ptrc-EngA (Figure 6A).

Cold sensitivity, reported for EngA-depleted strains of *E. coli* (Bharat and Brown, 2014), is presumably related to the temperature dependence of rRNA folding and to the role of EngA as rRNA chaperone in ribosome assembly and should thus be a universal property, observed whenever EngA activity is compromised. With the purpose of getting further insights into cyanobacterial EngA and its functional connection with PipX, we compared in parallel the growth of control and mutant strains of *S. elongatus* at 30 and 20°C.

At 20°C, strains *engA*^–/+^ and 3^N^Ptrc-EngA grew, respectively, worse and slightly better than their corresponding controls, indicating that the function of EngA is even more important at low temperatures. In contrast, *pipX null* and 1^S^Ptrc-PipX grew better and worse, respectively, than their controls at 20°C, indicating that PipX is slowing down the growth of *S. elongatus* at a low temperature. In addition, 1^S^Ptrc-PipX/3^N^Ptrc-EngA grew better than 1^S^Ptrc-PipX at 20°C but not at 30°C, suggesting that EngA counteracts the negative impact of PipX on growth at a low temperature (Figures 6A,B).

To obtain additional evidence of gene interaction between *pipX* and *engA* in *S. elongatus*, we introduced the *engA* null allele into *pipX* and 1^S^Ptrc-PipX backgrounds and asked whether the absence or three- or fourfold higher levels of PipX altered the viability of *engA*^–/+^cultures. While single *engA*^–/+^ and double *pipX engA*^–/+^ mutants appeared equally affected, 1^S^Ptrc-PipX/*engA*^–/+^lost viability even faster than *engA*^–/+^, and the cell defects were more pronounced, indicating that PipX is particularly toxic under conditions in which EngA activity is compromised (Figures 6C,D).

In summary, genetic analysis shows a gene interaction between *pipX* and *engA*, particularly at the environmentally relevant temperature of 20°C, a condition where the EngA role as ribosome chaperon would be more important and where the *pipX* null mutant shows a growth phenotype.

### Overexpression of PipX Induces Bleaching and Impairs Viability

In previous studies, we focused on determining genetic contexts associated to PipX toxicity in *S. elongatus* by PCR analyses of the segregation of the corresponding *glnB or pipX* mutant alleles in different backgrounds or performing growth tests on plates (Espinosa et al., 2009, 2010, 2018; Laichoubi et al., 2012). To get further insights into the phenomenon of PipX toxicity and its possible connection with EngA, we now investigated the phenotypic impact of PipX overexpression in *S. elongatus* cultures in the presence of IPTG under otherwise standard growth conditions.

**FIGURE 7:**
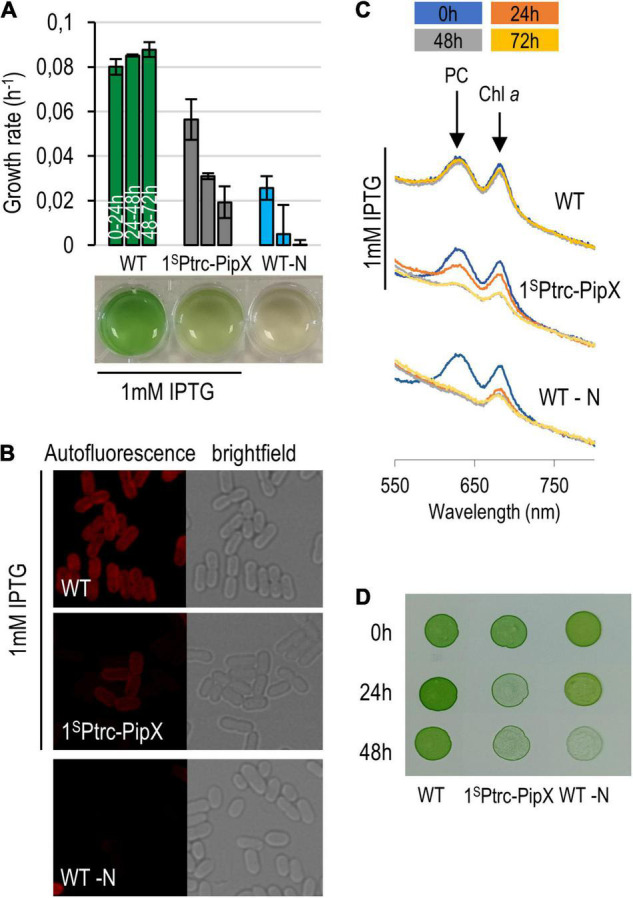
Phenotypic features of PipX overexpression. **(A)** Growth rate of 1^S^Ptrc-PipX (BG11) and wild-type (BG11 minus NaNO_3_) cultures and their appearance at the end of the experiment. **(B)** Micrographs (confocal and bright-field microscopy) from the same cultures at the 72-h point. **(C)** Absorbance spectra at 24-h intervals with indication of the phycocyanin and chlorophyll *a* peaks. **(D)** Recovery of cultures after transfer of the indicated cultures and given timepoints to BG11 plates. In all cases, IPTG was used only when indicated.

Overexpression of PipX at 1 mM IPTG has a severe impact on 1^S^Ptrc-PipX cultures. These slowed down growth and acquired a chlorotic appearance, indicating that they were degrading their photosynthetic pigments. Under these conditions, we observed no differences between 1^S^Ptrc-PipX and 1^S^Ptrc-PipX/3^N^Ptrc-EngA cultures (data not shown), probably due to the high PipX/EngA ratio. Since overexpression of PipX resembled the behavior of *S. elongatus* subjected to nutrient stress, we compared 1^S^Ptrc-PipX cultures subjected to PipX overexpression with WT cultures subjected to nitrogen deprivation (-N). As shown in Figure 7A, the growth rate of PipX overexpressing cultures decreased significantly but was slower than that of the nitrogen-starved cultures. Chlorosis was also slower, and at 72 h, only the -N cultures were completely bleached (Figure 7A). Confocal microscopy showed a reduced autofluorescence signal after excitation with a 633 nm laser, and all chlorotic cells showed a normal cell morphology (Figure 7B). In line with this, the spectrophotometric analysis of pigment composition showed that PipX overexpression results in a slower (approximately 24-h delay) but similar reduction of the 680 nm (Chla) and 620 nm (phycocyanin) peaks in comparison to the nitrogen-deprived culture (Figure 7C).

Importantly, cell viability (Figure 7D) decreased comparatively faster than growth rate and pigment loss in PipX overexpression cultures than in -N cultures, indicating that intracellular PipX accumulation induces chlorosis but not the complex and adaptative response associated to the same phenomenon triggered by nitrogen starvation in *S. elongatus*.

In a recent work from T. Omata laboratory (Sakamoto et al., 2021), the phenotypic consequences of uncontrolled PipX were addressed in a *glnB* null mutant rescued by supplementation of the anti-oxidant α-tocopherol. Interestingly, impaired electron transfer in both the reducing and oxidized sizes of Q_A_ and disturbance of the PSII repair mechanisms were observed in the single *glnB* mutant and not in the double *glnB pipX* mutant. Therefore, increasing the levels of “free” PipX by either counteracting (partially) its lethal effects in a *glnB* background or overexpressing the protein, as shown here, results in impairment of both photosynthesis (*via* pigment degradation) and viability.

The mechanism by which an excess of PipX triggers chlorosis and growth retardation is unknown. At least two transcriptional regulators are known to activate the *nblA* gene to trigger the degradation of the light-harvesting antenna (phycobilisome) in *S. elongatus*, NtcA and NblR (Luque et al., 2002; Salinas et al., 2007; Ruiz et al., 2008). NblR, probably involved in the downregulation of photosynthetic electron transport during nutrient starvation, controls processes which are critical for viability during starvation (Schwarz and Grossman, 1998). Given the PipX role as NtcA coactivator, its overexpression would activate *nblA* and other NtcA-dependent genes to induce chlorosis but would not affect NblR-dependent targets required for maximal *nblA* expression and recovery from chlorosis.

Since EngA has been implicated in the photosystem II repair cycle in *A. thaliana* thylakoids (Kato et al., 2018), the finding that PipX is specifically slowing down growth during cold stress, where ROS production increases because the light reaching the photosystems exceeds the energy requirement for metabolism, is very intriguing. The possibility that PipX, a protein conserved and only present in cyanobacteria, may have a role in acclimation to photosynthesis-induced redox stress by modulating EngA activity is therefore worth exploring.

### A Model for PipX Regulation of EngA

**FIGURE 8:**
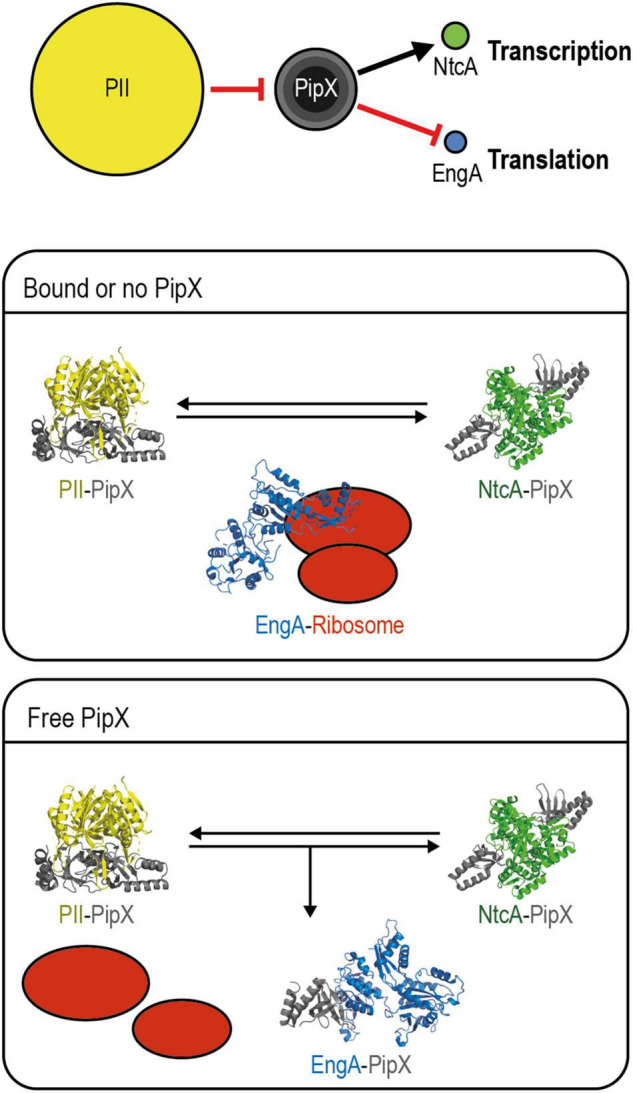
Model for PipX regulation of EngA. Top panel: schematic representation of the discussed signaling pathway from PII to NtcA and EngA with indication of the relevant protein–protein interactions and alternative scenarios for PipX–EngA interactions. The encircled areas are proportional to protein abundance (PII trimers; PipX: trimers, dimers, and monomers from inner to outer circles, respectively; NtcA dimers; and EngA monomers). Red hammerhead and black arrows indicate inactivation by sequestration and co-activation, respectively. Middle panel, “bound or no PipX”: under standard culture conditions, PipX is involved in partner swapping with PII and NtcA, while EngA is active. Bottom panel, “Free PipX”: when the level of free PipX rises, it also binds to EngA, interfering with its ribosome assembly function. PDB models: PII–PipX from 2XG8; NtcA-PipX from 2XKO; closed EngA predicted with SWISS model using as query *Synechococcus elongatus* protein sequence based on the PDB 4DCS model; open EngA from *Escherichia coli* model 3J8G (see text for additional details).

To integrate previous information on PipX with the results presented in this work, we propose a simple and speculative regulatory model which is summarized in Figure 8. Additional research is now aimed to gain insights into the molecular details and additional players involved.

Consistent with their roles as hubs of the nitrogen interaction network, PII and PipX are very abundant regulatory proteins. Importantly, PII is in a 14-fold excess over PipX, which is in a roughly similar excess over its regulatory targets NtcA and EngA, according to data previously reported by us and others (Guerreiro et al., 2014; Labella et al., 2016). In this scenario, PipX–NtcA and PipX–EngA complex formation would depend on the levels of PII effectors, determining the availability of “free” PipX. In addition, the physiological context in which PipX and EngA interact in cyanobacteria must greatly depend on the availability of EngA effectors, just as the formation of PipX–NtcA complexes depend on the availability of the NtcA effector 2-OG. For simplicity, the different environmental or genetic contexts in which PipX–EngA complexes are, respectively, favored or not are referred to as “free PipX” or as “bound or no PipX” in the model, and we assume that the affinity of PipX for EngA is lower than for NtcA.

Notwithstanding the opposite effects of PipX binding on the function of these two targets, stimulatory for NtcA and presumably inhibitory for EngA, there is an obvious parallelism between the regulation of NtcA and EngA activities controlled by the sequestration of PipX by PII in response to different combinations of effector molecules and affecting gene expression, each one at a different level. The NtcA pathway branch involves 2-OG-dependent transcription of the large nitrogen assimilation regulon, and the EngA one involves guanine nucleotide-dependent ribosome assembly, and here it is tempting to propose that the interference of PipX with EngA function would have an impact on the translation of a relatively large subset of cyanobacterial genes, perhaps inducing adaptation to adverse conditions such as cold stress.

In summary, we propose that PipX binds to EngA to slow down growth under conditions in which the levels of PII and EngA effectors, together signaling energy status and C/N ratios, allow the formation of a significant number of PipX–EngA complexes and interfere with ribosome assembly. This situation, which can be achieved by genetic manipulation resulting in increases of the intracellular PipX/PII ratio, would occur at the environmentally relevant temperature of 20°C and probably under other non-optimal conditions.

## Concluding Remarks

We have shown by BACTH *in vitro* and genetic analyses that (a) there are both physical and functional connections between PipX and EngA, two of the six nodes of the (default) PipX synteny network, (b) the main interaction determinants map to the TLD/KOW and GD1 domains of the proteins, (c) *engA* is essential, and lowering its gene dosage causes cold sensitivity as well as severe and pleiotropic effects in *S. elongatus*, (d) EngA overexpression is well tolerated, with no apparent phenotypic consequences, (e) there is gene interaction between *pipX* and *engA* under conditions that are relevant for both the EngA role as ribosome chaperon and cyanobacterial adaptation to key environmental factors such as temperature, and (f) *in vivo* results are compatible with EngA being the essential PipX target involved in PipX toxicity.

This work expands the PII–PipX interaction network by addition of the essential ribosome assembly GTPase EngA. This new PipX interactant is, so far, the best candidate for the long-time-predicted low-affinity binding partner of PipX involved in the intriguing phenomenon of PipX toxicity. Future work is aimed to understand the molecular details and physiological context of the PipX–EngA interaction.

## Data Availability Statement

The original contributions presented in the study are included in the article/Supplementary Material, further inquiries can be directed to the corresponding author/s.

## Author Contributions

PS, RC, JE, JL, and AC designed the research. CJ, PS, AL, RC, JE, and JL performed the research. AC wrote the manuscript. All authors analyzed the data, contributed to manuscript revision, and read and approved the submitted version.

## Conflict of Interest

The authors declare that the research was conducted in the absence of any commercial or financial relationships that could be construed as a potential conflict of interest.

## Publisher’s Note

All claims expressed in this article are solely those of the authors and do not necessarily represent those of their affiliated organizations, or those of the publisher, the editors and the reviewers. Any product that may be evaluated in this article, or claim that may be made by its manufacturer, is not guaranteed or endorsed by the publisher.

## Footnotes

1
https://dfgm.ua.es/genetica/investigacion/cyanobacterial_genetics/dCLG/
