# Supplementary material for: Regulatory Connections Between the Cyanobacterial Factor PipX and the Ribosome Assembly GTPase EngA
Source: Front Microbiol. 2021 Dec 9;12:781760. doi: 10.3389/fmicb.2021.781760 (PMC8696166; doi:10.3389/fmicb.2021.781760)
Supplement: Supplementary file 1 [file Table_1.PDF]

Table S1. Construction of plasmids for BACTH analysis

Forward	Reverse	Enzymes	Cloned into	Plasmid	Fusion protein expressed
PipX-BTH-2F	PipX-BTH-2R	*Bam*HI + *Kpn*I	pKT25	pUAGC1047	T25:PipX
			pUT18	pUAGC934	PipX:T18
			pKTN25	pUAGC1045	PipX:T25
PipX-BTH-F	PipXresi70Rev	*Bam*HI + *Eco*RI	pUT18c	pUAGC1026	T18:PipX$^{1-70}$
			pKT25	pUAGC1027	T25:PipX$^{1-70}$
PipX-BTH-F	PipXresi54Rev	*Bam*HI + *Eco*RI	pUT18c	pUAGC1024	T18:PipX$^{1-54}$
			pKT25	pUAGC1025	T25:PipX$^{1-54}$
PipX-BTH-2F	PipX-BACTH-R70-2R	*Bam*HI + *Kpn*I	pUT18	pUAGC1095	PipX$^{1-70}$:T18
			pKTN25	pUAGC1104	PipX$^{1-70}$:T25
PipX-BTH-2F	PipX-BACTH-R54-2R	*Bam*HI + *Kpn*I	pUT18	pUAGC1096	PipX$^{1-54}$:T18
			pKTN25	pUAGC1103	PipX$^{1-54}$:T25
2340-BTH-1F	2340-BTH-1R	*Bam*HI + *Kpn*I	pUT18c	pUAGC920	T18:EngA
			pUT18	pUAGC1022	EngA:T18
			pKT25	pUAGC921	T25:EngA
			pKTN25	pUAGC1023	EngA:T25
2340-BTH-1F	GD1-BTH-1R	*Bam*HI + *Kpn*I	pUT18c	pUAGC1070	T18:GD1
			pKT25	pUAGC1071	T25:GD1
2340-BTH-1F	GD1-BTH-2R	*Bam*HI + *Kpn*I	pUT18	pUAGC1072	GD1:T18
			pKTN25	pUAGC1073	GD1:T25
2340-BTH-1F	EngA-BTH-2R	*Bam*HI + *Kpn*I	pUT18c	pUAGC1060	T18:GD1-GD2
			pKT25	pUAGC1061	T25:GD1-GD2
2340-BTH-1F	EngA-BTH-355sin-R	*Bam*HI + *Kpn*I	pUT18	pUAGC1062	GD1-GD2:T18
			pKTN25	pUAGC1063	GD1-GD2:T25
EngA-BTH-2F	EngA-BTH-4R	*Bam*HI + *Kpn*I	pUT18c	pUAGC1064	T18:GD2-KH
			pKT25	pUAGC1065	T25:GD2-KH
EngA-BTH-2F	2340-BTH-1R	*Bam*HI + *Kpn*I	pUT18	pUAGC1066	GD2-KH:T18
			pKTN25	pUAGC1067	GD2-KH:T25
NtcA-BTH-2F	NtcA-BTH-1R	*Xba*I + *Kpn*I	pUT18c	pUAGC1074	T18:NtcA
			pKT25	pUAGC1075	T25:NtcA
PII-BTH-F	PII-BTH-R	*Bam*HI + *Kpn*I	pKT25	pUAGC1048	T25:PII
PlmA-BTH-1F	PlmA-BTH-1R	*Bam*HI + *Kpn*I	pKT25	pUAGC1001	T25:PlmA
