# Supplementary material for: Regulatory Connections Between the Cyanobacterial Factor PipX and the Ribosome Assembly GTPase EngA
Source: Front Microbiol. 2021 Dec 9;12:781760. doi: 10.3389/fmicb.2021.781760 (PMC8696166; doi:10.3389/fmicb.2021.781760)
Supplement: Supplementary file 2 [file Image_1.PDF]

SUPPLEMENTARY

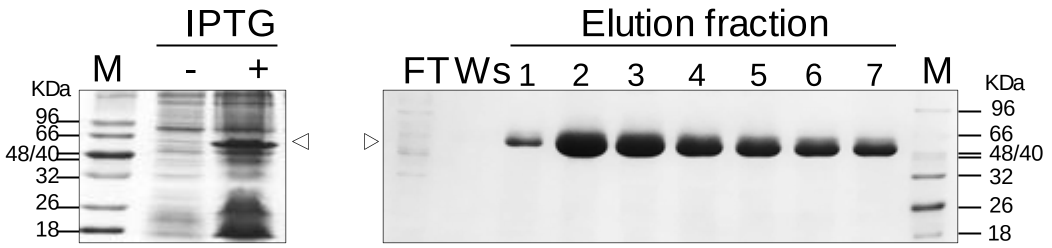

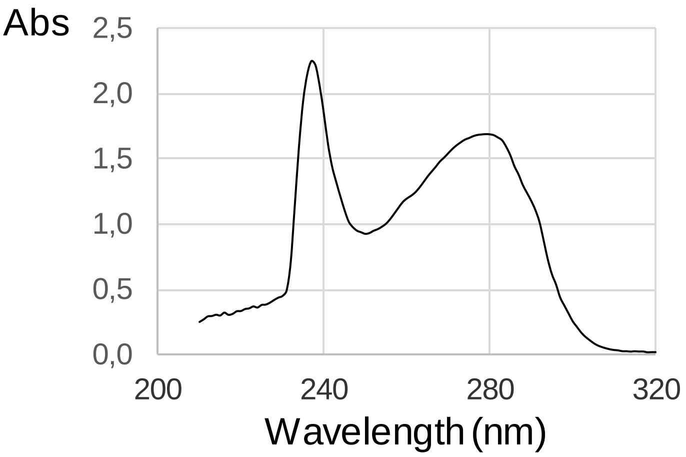

Figure S1. Purification of recombinant His-tagged EngA. SDS-PAGE analysis of *E. coli* BL21 cell extracts after IPTG induction (+) or no (-) of recombinant EngA. Soluble protein fraction was loaded on Ni-columns and flow-through (FT), wash (Ws) and elution fractions were recovered and electrophoresed. Gels were stained with Coomassie blue. Arrowheads show the position of His-EngA. M refers to the marker lane (NZY-tech low molecular weight protein marker). The absorbance spectra of diafiltrated and concentrated EngA fractions is shown.

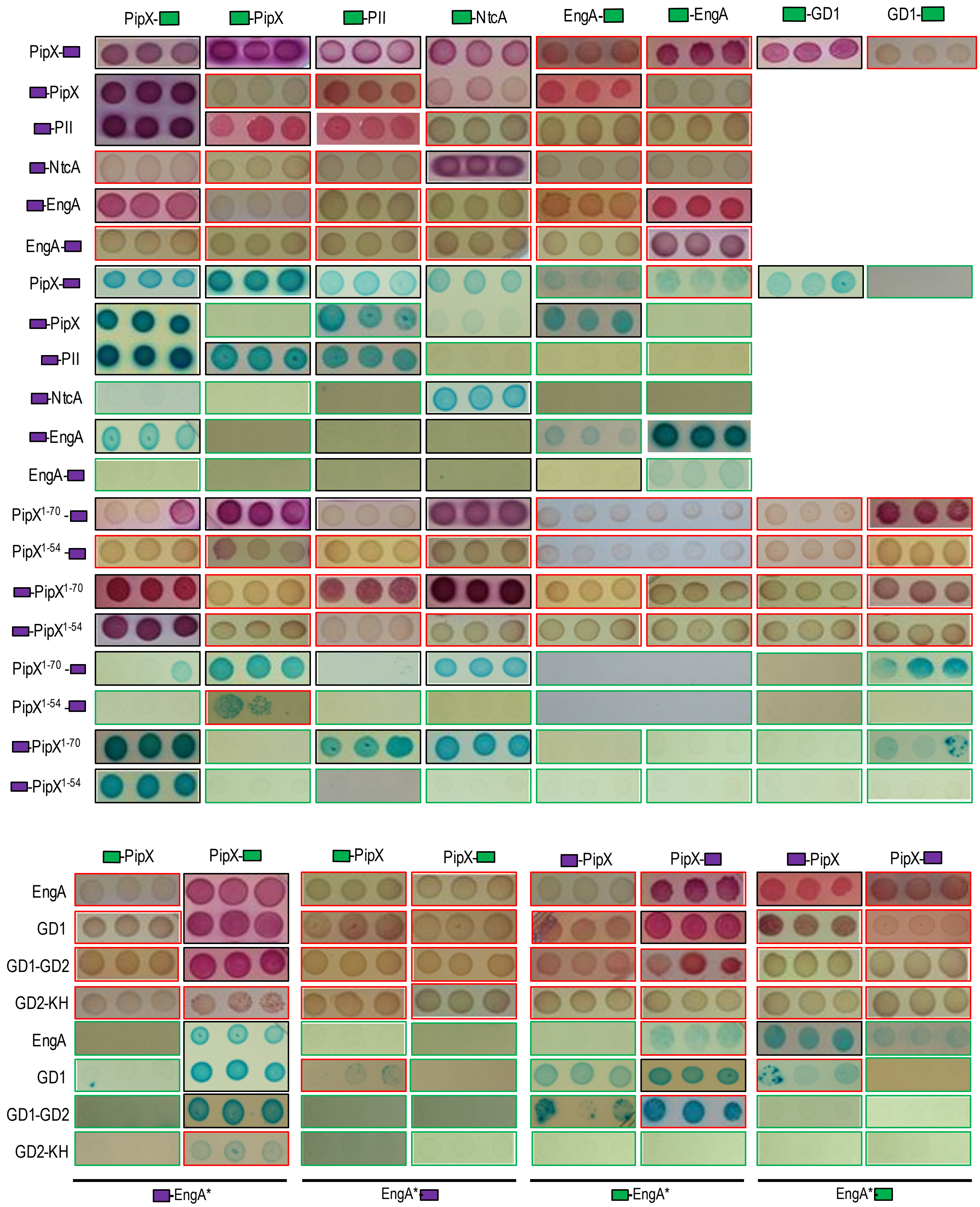

Figure S2. Representative photographs of BACTH assays used to produce the heatmaps of Figures 2-3. Pictures were taken at 24h, 48h or 72h (black, red or green outline).

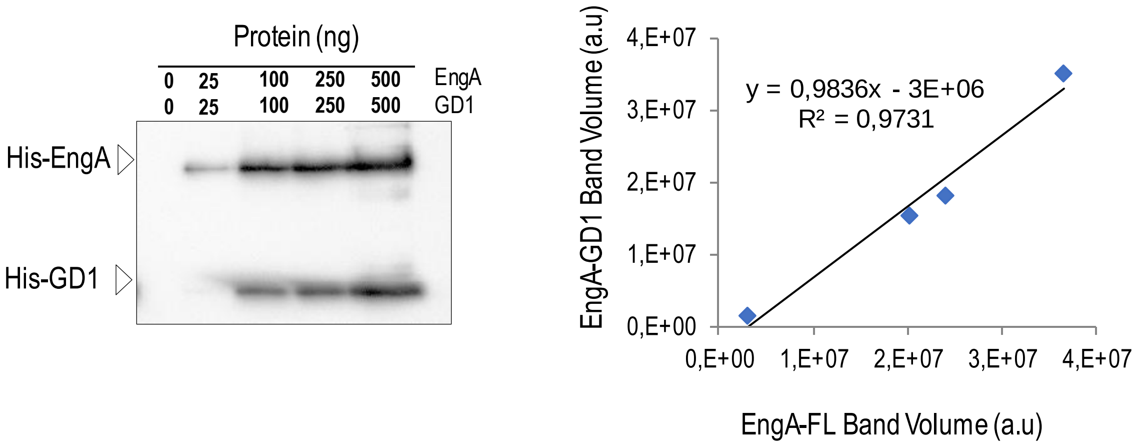

Figure S3. Immunodetection signals of His-EngA and His-GD1. Mixtures of His-EngA and His-GD1 were detected by Western-blot. The intensity of each band was subtracted using the empty lane indicated as 0. The band volumes (in arbitrary units) were plotted in the correlation graph together with the slope.
